# A Novel Lung Explant Model for the Ex Vivo Study of Efficacy and Mechanisms of Anti-Influenza Drugs

**DOI:** 10.4049/jimmunol.1402283

**Published:** 2015-05-01

**Authors:** Ben Nicholas, Karl J. Staples, Stefan Moese, Eric Meldrum, Jon Ward, Patrick Dennison, Tom Havelock, Timothy S. C. Hinks, Khalid Amer, Edwin Woo, Martin Chamberlain, Neeta Singh, Malcolm North, Sandy Pink, Tom M. A. Wilkinson, Ratko Djukanović

**Affiliations:** * Clinical and Experimental Sciences, University of Southampton Faculty of Medicine, Sir Henry Wellcome Laboratories, Southampton General Hospital, Southampton SO16 6YD, United Kingdom;; † Southampton National Institute for Health Research Respiratory Biomedical Research Unit, Southampton General Hospital, Southampton SO16 6YD, United Kingdom;; ‡ 3-V Biosciences, Menlo Park, CA 94025;; § Department of Cardiothoracic Surgery, Southampton General Hospital, Southampton SO16 6YD, United Kingdom; and; ¶ Department of Cellular Pathology, Southampton General Hospital, Southampton SO16 6YD, United Kingdom

## Abstract

Influenza A virus causes considerable morbidity and mortality largely because of a lack of effective antiviral drugs. Viral neuraminidase inhibitors, which inhibit viral release from the infected cell, are currently the only approved drugs for influenza, but have recently been shown to be less effective than previously thought. Growing resistance to therapies that target viral proteins has led to increased urgency in the search for novel anti-influenza compounds. However, discovery and development of new drugs have been restricted because of differences in susceptibility to influenza between animal models and humans and a lack of translation between cell culture and in vivo measures of efficacy. To circumvent these limitations, we developed an experimental approach based on ex vivo infection of human bronchial tissue explants and optimized a method of flow cytometric analysis to directly quantify infection rates in bronchial epithelial tissues. This allowed testing of the effectiveness of TVB024, a vATPase inhibitor that inhibits viral replication rather than virus release, and to compare efficacy with the current frontline neuraminidase inhibitor, oseltamivir. The study showed that the vATPase inhibitor completely abrogated epithelial cell infection, virus shedding, and the associated induction of proinflammatory mediators, whereas oseltamivir was only partially effective at reducing these mediators and ineffective against innate responses. We propose, therefore, that this explant model could be used to predict the efficacy of novel anti-influenza compounds targeting diverse stages of the viral replication cycle, thereby complementing animal models and facilitating progression of new drugs into clinical trials.

## Introduction

Influenza has a major impact on global health, especially during seasonal epidemics, resulting in significant mortality, particularly among children and the elderly (1). It also causes serious complications in patients with chronic respiratory diseases and in immunosuppressed individuals (2). Despite significant resources spent on preventing and treating influenza, there remains a large unmet need for effective anti-influenza virus therapies. Although recommended by the World Health Organization for at-risk populations (3, 4), vaccination against influenza is not fully effective. Drugs targeting the viral neuraminidases, such as oseltamivir (Tamiflu) and zanamivir (Relenza), and M2 ion channel inhibitors, such as Amantadine, are showing increased resistance (5, 6). Furthermore, their effectiveness has not been definitively proven in patients with chronic airways diseases in whom the impact of influenza on morbidity and mortality is higher than in the general population (7).

The limited size of the viral genome restricts the scope of therapeutic development targeting influenza viral proteins. Recent developments in technology to discover novel host gene targets, such as genome-wide small interfering RNA and homozygous gene perturbation screens (8–13), have identified a large number of genes involved in the replication of the influenza virus that are candidate targets (14). Progression of therapeutics identified through such screening requires additional proof of efficacy before embarking on clinical trials in human volunteers.

Preclinical testing of influenza therapeutics has been restricted to a few animal species, such as ferrets, which can be infected by strains that also affect humans (15); however, their use in the development of drugs, especially those targeting human host defenses, is limited by interspecies differences in gene sequence, protein structure, and also potential differences in viral–host interactions.

The difference in inflammatory responses to viral infection between therapies that target early and late viral life cycle replication events has not been fully investigated in humans. This is partly because existing cell models do not produce the wide range of inflammatory mediator responses observed in human infections, and partly because of challenges associated with measuring mediator responses in biofluids derived from in vivo experimental infections of human volunteers.

To address the current limitations in development of anti-influenza drugs, we have developed a preclinical testing platform in which lung tissue samples are infected ex vivo with influenza virus. The extent of infection of lung tissue is then quantified by flow cytometry, and inflammatory responses are assessed by measuring proinflammatory mediator production secreted by the infected tissue. We report in this work on the value of this explant model by comparing the antiviral efficacy of targeting viral entry mechanisms to inhibit replication using a vATPase inhibitor with that of a neuraminidase inhibitor (oseltamivir) that inhibits viral shedding. We discuss the potential benefits of such a model in determining infection characteristics and therapeutic responses in patients with chronic lung diseases.

## Materials and Methods

### Study design

We first optimized the methods for identifying and quantifying influenza infection in cells and tissues by flow cytometry. The lung explant model was then validated by quantifying the extent of epithelial cell infection and viral shedding from bronchial biopsies obtained by bronchoscopy. The dose of infection (multiplicity of infection [MOI]) required was then compared with that needed to infect standard monolayer primary bronchial epithelial cell (PBEC) cultures. The two culture models were compared further in respect of inflammatory responses by measuring a set of cytokines/chemokines, many of which have been previously shown to be modulated in vivo during human influenza infection (16). The explant model was then applied to study the antiviral effects of a vATPase inhibitor, TVB024, which inhibits viral entry into epithelial cells, and oseltamavir, a neuraminidase inhibitor that inhibits virus release from infected epithelial cells.

#### Materials.

A/H3N2/X31 and A/H3N2/Wisconsin/67/2005 seed stocks were obtained from the National Institute for Biological Standards and Control propagated in embryonated specific pathogen-free chicken eggs, and, subsequently, purified from egg allantoic fluid by sucrose density gradient ultracentrifugation (Virapur LLC, San Diego, CA). Stock viral titer was determined by Madin-Darby canine kidney plaque assay using standard protocols. Anti-influenza nuclear protein mAb conjugated to FITC was purchased from BD Biosciences (Cowley, Oxford, U.K.). Rabbit polyclonal anti-A/H3N2/Wisconsin/67/2005 Ab was produced from UV-inactivated virus by Eurogentec (Seraing, Belgium). Oseltamavir carboxylate and the vATPase inhibitor TVB024 were synthesized in-house, and their purity was confirmed to be >99% by nuclear magnetic resonance and mass spectrometry. Collagenase type I from *Clostridium histolyticum* was purchased from Sigma-Aldrich (Poole, Dorset, U.K.). Abs used for flow cytometry and immunohistochemistry were purchased from BD Biosciences (Cowley, Oxford, U.K.), Abcam (Cambridge, U.K.), and Invitrogen (Paisley, Scotland, U.K.).

### Lung tissue and cell culture

To develop the infection model, lung tissue and cultured primary cell lines were required, including epithelial and endothelial cells, fibroblasts, and leukocytes.

Bronchial lung tissue and epithelial cells for ex vivo infection were obtained by fiberoptic bronchoscopy from 10 healthy volunteer subjects with no evidence of airways disease, as shown by normal lung function spirometry tests and also no evidence of reversibility to the β_2_-agonist bronchodilator salbutamol. All subjects were nonatopic, as shown by negative skin prick tests to a panel of common aeroallergens. Standard research protocols were applied (17, 18), and up to 10 bronchial biopsy samples and 6 bronchial brushings were taken from the subcarinae of the proximal bronchi. Pairs of bronchial explants were rested overnight in 500 μl complete AIM-V medium supplemented with penicillin and amphotericin, as previously described (19), and the medium was exchanged prior to infection.

Resected human lung tissue was obtained from patients undergoing surgical lobectomy for lung cancer at Southampton University Hospital. Parenchymal tissue from donors without evidence of obstructive lung disease or asthma, distant from the resection margin and any gross pathology, was dissected and the tissue was then dissected into small cubes (1–3 mm^3^) and transferred to 24-well culture dishes, three pieces per well.

Both the PBECs and lung tissue explants were cultured in a humidified tissue culture incubator at 37°C, 5% CO_2_.

### Immortal cell line culture

The immortalized type II alveolar cell line A549 (ATCC CCL-185) was cultured in DMEM supplemented with 10% (v/v) heat-inactivated FCS, nonessential amino acids, and l-glutamine. Cells were used from logarithmically growing cultures at up to 90% confluence.

### Primary cell line culture

A number of primary cell cultures was first established for optimization of the flow cytometric analysis of bronchial biopsies dispersed postinfection (see below).

Primary human lung fibroblasts were isolated by outgrowth from human bronchial biopsies (20). Cells were maintained in DMEM supplemented with glutamine, penicillin-streptomycin, sodium pyruvate, and nonessential amino acids and used within passages 1–2. HUVECs were isolated, as previously described (21), and were a gift of T. Millar (University of Southampton). They were maintained in DMEM supplemented with 10% human AB serum on gelatin-coated culture surfaces and used within passages 1–2. PBECs were cultured from bronchial brushings as monolayers (22). They were maintained on collagen I–coated culture surfaces in bronchial epithelial growth medium (Lonza) and used within passages 1–2.

All adherent cell cultures were isolated from monolayers using trypsin-EDTA just before use and resuspended at 1 × 10^6^ cells/ml in PBS prior to mixing for flow cytometric analysis.

Human PBMCs were obtained from venous blood by density centrifugation using Lymphoprep, as described in the manufacturer’s instructions. PBMCs were resuspended in PBS at a concentration of 1 × 10^6^/ml.

For infection experiments, PBECs were cultured in 24-well tissue culture plates at a density of 0.5 × 10^4^ cells/well, and, 24 h prior to infection, media were changed to basal growth medium supplemented with insulin, transferrin, and selenium solution (Life Technologies, Paisley, U.K.).

### Infection of epithelial cells and lung tissue explants

The A549 cells, PBECs, and lung tissue samples were treated with drugs (oseltamivir or vATPase inhibitor) or 0.05% (v/v) DMSO carrier control (where appropriate) in their respective media for 2 h prior to infection. Influenza virus (A/H2N3/X31 or A/H3N2/Wisconsin/67/2005, as appropriate) was added in the presence of inhibitor or control and incubated for an additional 2 h. Virus was then added at MOI of 0.2 to monolayer cell cultures, and at log 7.4 infectious unit dose to lung explant tissues. Samples were then washed three times by media replacement to remove excess virus and incubated for further time points in complete AIM-V or basal growth medium for explant and PBEC cultures, respectively, with the addition of study drugs or DMSO control. At appropriate time points, culture medium samples were collected and centrifuged at 400 × *g*, 4°C for 5 min to remove cellular material. Samples were analyzed by multiplexed ELISA for a set of cytokines and chemokines (Mesoscale Discovery, Rockville, MD).

### Assessment of endosomal acidification using acridine orange assay

The effect of TVB024 on endosomal acidification was assessed by acridine orange staining for determination of endosomal pH. Briefly, A549 cells were preincubated with TVB024 for 16 h. The culture media were then replaced with HBSS containing 5 μg/ml acridine orange (A1301; Life Technologies) for 15 min at 37°C. The cells were washed three times with HBSS and then incubated with HBSS containing the drug at the same initial concentrations. The culture plates were then immediately assessed for acridine orange staining using a spectrophotometer equipped with a long pass filter set (479/28 nm excitation/>500 nM emission).

#### Flow cytometric analysis of infection.

Postinfection with virus, the PBECs were dispersed with trypsin-EDTA, which was subsequently neutralized using 10% (w/v) FCS and removed by centrifugation at 400 × *g* for 5 min. The bronchial explants were dispersed with 1 mg/ml collagenase in basal RPMI 1640 culture medium for 90 min at 37°C, and the cells were then centrifuged at 400 × *g* for 5 min to remove collagenase. Dispersed explant and PBECs were resuspended in flow cytometry buffer (0.5% w/v BSA, 2 mM EDTA in Dulbecco’s PBS) and treated for 10 min with 5 mg/ml human IgG on ice prior to extracellular staining with fluorochrome-labeled mAbs for 30 min on ice. For the analysis of dispersed biopsies Abs against CD45 (pan-leukocyte marker), CD90/Thy1 (fibroblast marker), CD326/EpCAM (epithelial cell marker), and CD34 (endothelial/hematopoietic progenitor cell marker) were used to demonstrate the specificity of each of the markers for different structural and leukocyte cell populations. Thereafter, a combination of CD326 and CD45 Abs was used routinely to quantify infection of epithelial cells in bronchial biopsies. The cells were then fixed and permeabilized using BD Fix/Perm reagents prior to staining for intracellular expression of viral nucleoprotein using mAbs conjugated to FITC. Flow cytometry was performed using a FACSAria (BD Biosciences) with appropriate filters and settings. Data were analyzed using FACS Diva software.

### Immunohistochemistry

To validate the flow cytometric detection of virus infection and localize the infection in the lung tissue, immunohistochemistry was performed using samples fixed in acetone containing protease inhibitors overnight at −20°C. Samples were then embedded in glycol methacrylate, as previously described (23). The 2-μm sections were cut and applied to poly-*l*-lysine–coated glass slides. Staining was performed, as previously described (24), using the ABC system with peroxidase-linked secondary Abs against mouse or rabbit primary Abs. The same Ab clones were used for both immunohistochemistry and flow cytometry, following optimization of each Ab concentration for the relevant method. HRP signal was detected with diaminobenzidine stain, and with a hematoxylin counterstain. Images were captured using a Zeiss Axioskop2 microscope fitted with an Axiocam, using Zeiss KS400 v3.0 image analysis software. X31 virus infection was detected using unconjugated HB-65 mAb.

### Virus release assays

Due to the risk of highly potent antiviral compounds interfering with standard plaque assay, and the additional presence of infection-inhibitory proteins in the AIM-V culture medium used in explant cultures, a viral immunoassay validated against plaque assay was used to quantify viral shedding. Viral titer quantified by the two methods was log transformed to normalize the data prior to comparison of the differences in measurements using Bland-Altman analysis (Supplemental Fig. 3A), showing good agreement between both methods, with a mean difference of −0.005 (confidence interval, −0.106 to 0.096), well within the limits of agreement of 0.636–0.627.

PBECs and explant conditioned media were transferred to nitrocellulose membrane using bio-dot apparatus (Bio-Rad, Hemel Hempstead, U.K.). A standard curve of viral protein was generated using viral stocks of known infectious dose (determine by Madin-Darby canine kidney plaque assay) diluted in the same manner. Immobilized viral proteins were detected using rabbit polyclonal Abs specific for the influenza virus and visualized by chemiluminescence detection using anti-rabbit detection system (Bio-Rad). Signals were detected, and data were interpolated from the standard curves using a Versadoc chemiluminscence reader with QuantityOne software (Bio-Rad).

### Lactate dehydrogenase toxicity assay

A total of 25 μl tissue-conditioned medium was diluted in 100 μl phenol red-free DMEM, and then 25 μl assay buffer (Cytotox nonradioactive lactate dehydrogenase [LDH] assay; Promega, Madison, WI) was added to each well. Positive controls for each tissue donor were generated by sonicating tissue samples from control wells in their respective media for three bursts of 5 s at 7 μm on ice using a probe sonicator (Soniprep 150). Following addition of the assay buffer, the color was left to develop for 2 h, and then the plate was analyzed by measuring absorbance at 590 nm. Toxicity was measured as the percentage of LDH release relative to positive (100%) controls.

### Measurement of cytokines by multiplexed ELISA

Cell-free culture supernatants were analyzed by multiplexed ELISA (Mesoscale Discoveries), according to the manufacturers’ instructions, using a bespoke 8-plex ELISA for IL-6, IL-1β, TNF-α, IFN-γ, and chemokines IP-10, MCP-1, MIP1β, and IL-8. Briefly, conditioned media samples were diluted 1:1 in the supplied buffer containing carrier protein and applied to ELISA plates precoated with Abs directed against the eight mediators. A detection Ab solution prediluted in blocking buffer was applied, and the signal was analyzed on a SECTOR 2400 MSD plate reader. Data were analyzed using MSD discovery workbench software. Cytokine quantities were interpolated from standard curves generated using mixtures of recombinant proteins.

#### Data analysis and statistics.

Data were analyzed using paired nonparametric paired *t* test or nonparametric ANOVA with Dunn’s adjustment for multiple comparisons as appropriate to data distribution, using GraphPad Prism software (v6).

#### Ethics approval.

The collection of tissues was undertaken with written informed consent and was approved by and performed in accordance with the ethical standards of the Southampton and South West Hampshire Research Ethics Committee, LREC 09/H0504/109.

## Results

### Identification of structural cells within bronchial explant tissue using flow cytometry

**FIGURE 1.**
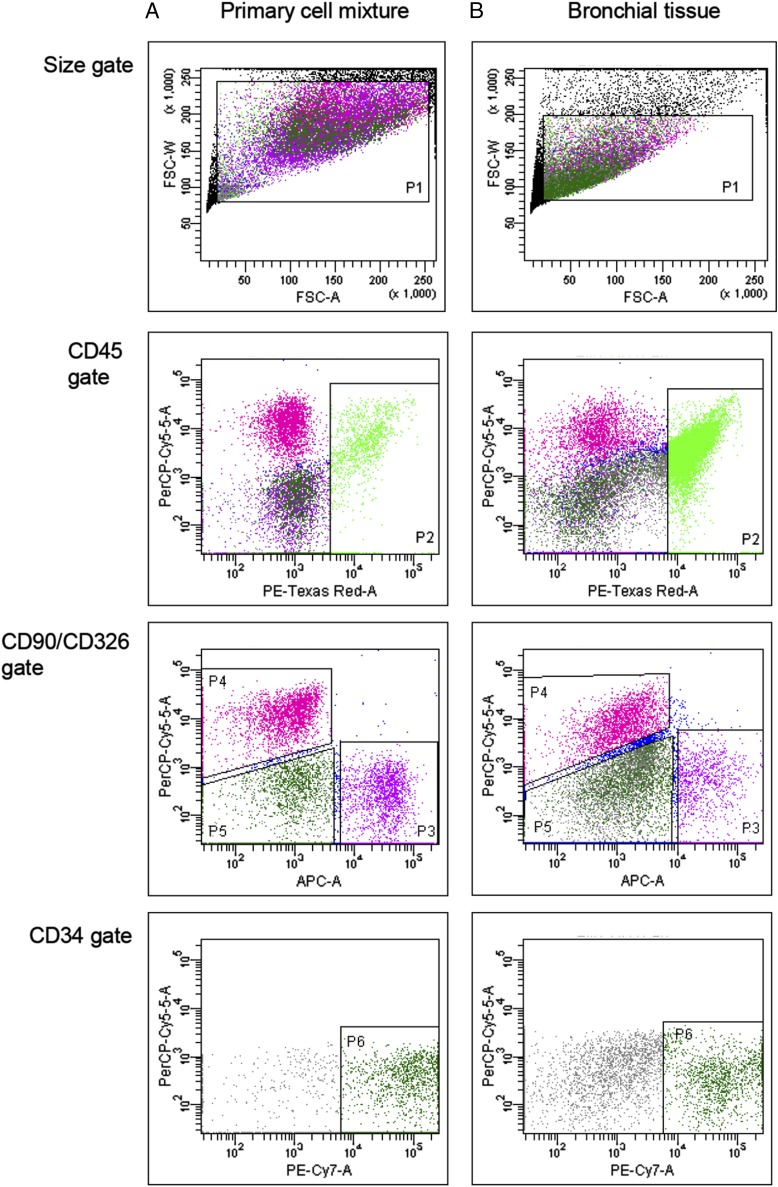
Detection of structural cells and leukocytes by flow cytometry. Gating strategy to detect CD45, CD90, CD326, and CD34 positive staining in the following: (**A**) equal mixtures of primary cultures of human bronchial epithelial cells, HUVECs, pulmonary fibroblast cells, and PBMCs; (**B**) enzymatically dispersed cells from bronchial tissue using the same gating strategy. Cell suspensions were stained for flow cytometry using Abs directed against CD90, CD326, CD34, and CD45 labeled with allophycocyanin, PerCPCy5.5, PE-Cy7, and PE-AF610, respectively, as described in *Materials and Methods*. Controls for each stain were prepared using appropriate isotype Abs labeled with the same fluorophores. Gating strategy applied: size gate to include single cell events (P1); identification of leukocytes using CD45 (P2); and excluding CD45 positive cells, fibroblasts (CD90^+^, P3) and epithelial cells (CD326^+^, P4) were identified. Excluding all other markers, endothelial cells stained positive for CD34 (P6). Clear populations of leukocytes, epithelial, fibroblast, and endothelial cells were observed when stained with Abs specific for each cell type.

We have previously used flow cytometry to quantify and characterize leukocyte populations within bronchial explant tissues (19, 25–27). To analyze structural cells (epithelial, fibroblast, and endothelial cells) within this tissue type, we optimized their detection using mAbs directed against surface markers unique to individual cell types. Specificity of the markers was confirmed first by immunohistochemistry (Supplemental Fig. 1). Flow cytometry was then applied to a mixture of primary cultures of cell types known to reside in bronchial tissue (Fig. 1A) or to enzymatically dispersed bronchial explant tissue (Fig. 1B). Primary cell culture resulted in increased cell volumes when compared with cells freshly dispersed from lung tissues, which reflected in elevated forward scatter properties (P1); however, comparable cell populations of leukocytes, fibroblasts, and epithelial and endothelial cells were observed. The results confirmed our ability to identify epithelial cells from within the tissue using flow cytometry using a gating strategy that excluded other cell populations such as leukocytes and fibroblasts.

### Pattern of influenza infection in bronchial explants

**FIGURE 2.**
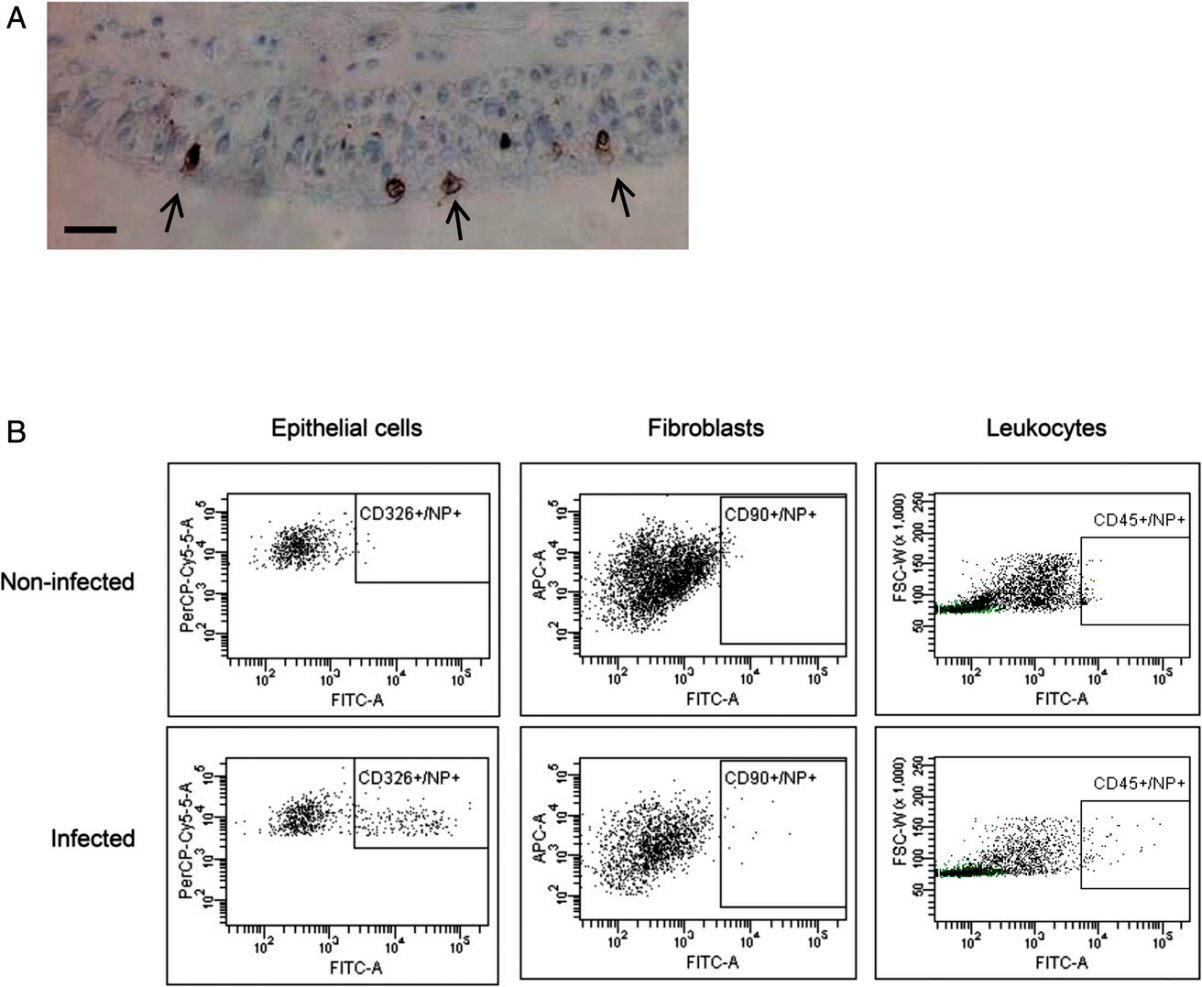
Identification of ex vivo influenza infection. Bronchial explants were infected with log7.4 infectious unit dose of A/H2N3/X31 influenza virus for 2 h and further incubated for a total of 24 h. (**A**) Immunohistochemical staining of a bronchial biopsy for viral infection using antiviral nuclear protein mAb (brown) and hematoxylin counterstain (blue) in bronchial tissues. Arrows indicate infected epithelial cells. Size bar indicates 50 μm. (**B**) Flow cytometric analysis of enzymatically dispersed bronchial biopsies to identify infected cells. Leukocytes were identified as CD45^+^ cells, epithelial cells were identified as a CD45^−^/CD326^+^ population, and fibroblasts were identified as CD45^−^/CD90^+^ population. Influenza infection in each cell type was measured by staining using mAbs directed to influenza nucleoprotein (NP), conjugated to FITC. Images shown are representative of three separate experiments. INF, infected; NI, not infected.

Infection of bronchial explants with influenza virus ex vivo was identified easily by intracellular expression of viral nucleoprotein within the epithelial cell population, both by immunohistochemistry and flow cytometry of cells. Immunohistochemistry showed infection only in the apical epithelial cells (Fig. 2A), visible as intense cytoplasmic and membrane staining. Similarly, infection was observed by flow cytometry largely in the epithelial cells and only rarely in leukocytes and fibroblasts (Fig. 2B).

### Comparison of PBEC and bronchial explant inflammatory responses

**FIGURE 3.**
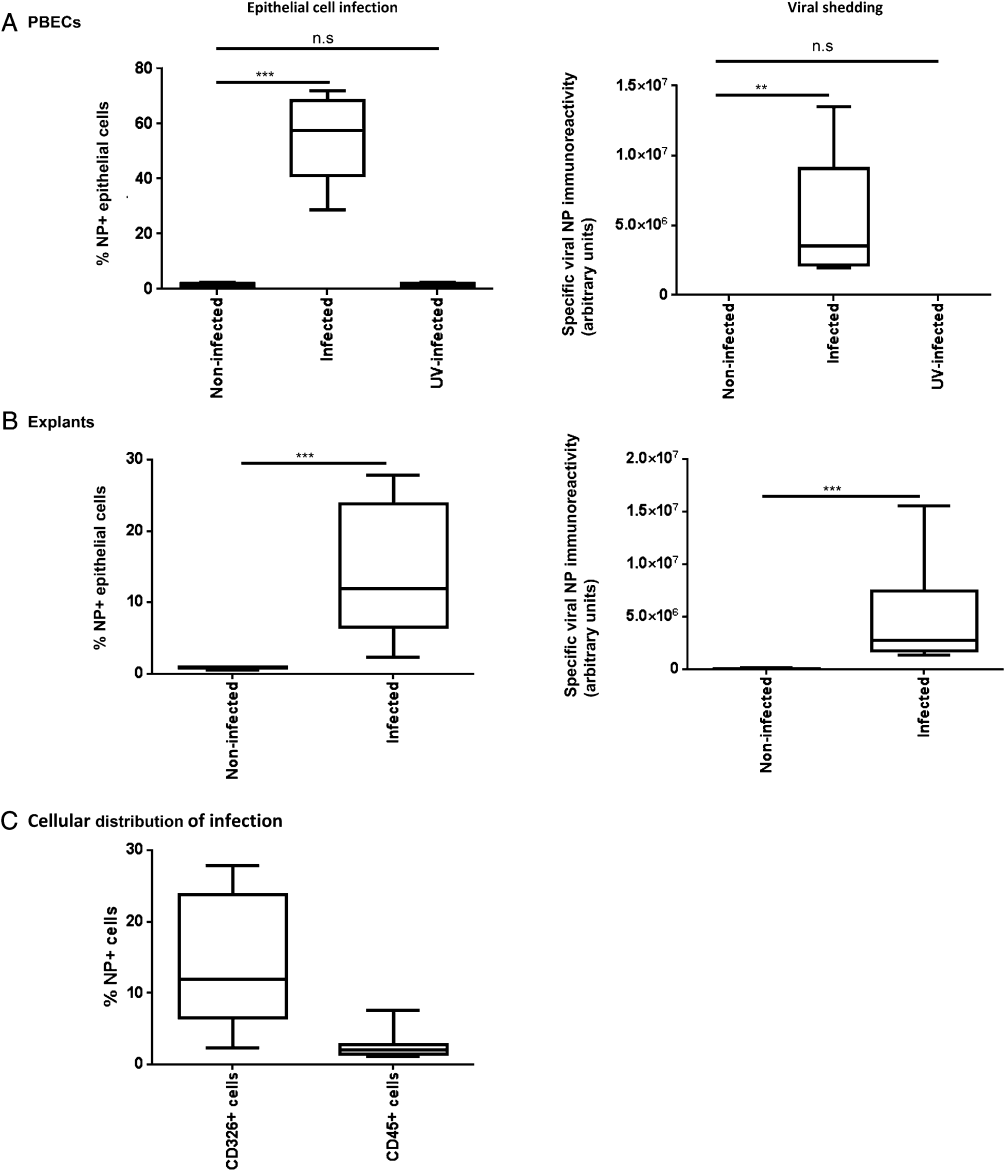
Quantification of influenza A infection in PBECs and bronchial explants. PBECs and bronchial explants were infected with a MOI of 0.2, and log7.4 IUD A/H3N2/Wisconsin/68/2005 influenza virus, respectively, for 2 h, as described in *Materials and Methods*, incubated for a maximum of 48 h, and then enzymatically dispersed. Cells were then stained for viral NP, as previously described, and analyzed by flow cytometry to identify NP^+^ epithelial cells. Infection in PBECs (**A**) and bronchial explants (**B**) was expressed as % NP^+^ epithelial cells. Viral shedding into the culture medium from infected PBECs and explants is expressed as arbitrary units of specific immunoreactivity to viral nuclear protein (background due to culture medium subtracted from data). (**C**) Influenza infection of human bronchial explants was observed principally in epithelial cells (CD45^−^/CD326^+^/NP^+^), with only limited infection of leukocytes (CD45^+^/NP^+^). Box and whisker plots indicate median and interquartiles ± range, *n* = 9 PBECs, *n* = 10 bronchial explants. **p < 0.01, ***p < 0.001.

PBECs infected with a MOI of 0.2 resulted in a median infection of 55% after 24 h of culture (Fig. 3A). No immunoreactivity was observed in cells exposed to UV-inactivated virus, demonstrating the specificity of this method for actively replicating intracellular virus. The virus dose for bronchial explants was optimized in dose-response experiments using resected lung parenchyma tissue samples, which were more freely available and of similar weight to bronchial explants used in subsequent analyses. The maximum infection rate in explants was 10% of epithelial cells at log 7.4 infectious unit dose in lung parenchyma (data not shown), an infection rate that could not be increased by adding more virus. This dose similarly resulted in an epithelial cell infection rate of 12% in bronchial explants (Fig. 3B). As epithelial numbers could only be calculated postinfection, by back calculation we calculated that the application of this maximum virus dose resulted in an approximate MOI of 200 for bronchial biopsies. This difference in MOI demonstrated key differences in viral handling and susceptibility between monolayer cultures and intact tissues. Infection was accompanied by release of viral proteins into the culture medium of both monolayer cells and lung tissues after 24 h of infection (Fig. 3A, 3B), suggesting active viral replication and specific release of virus. No viral shedding was observed in monolayer cells exposed to UV-inactivated virus.

**FIGURE 4.**
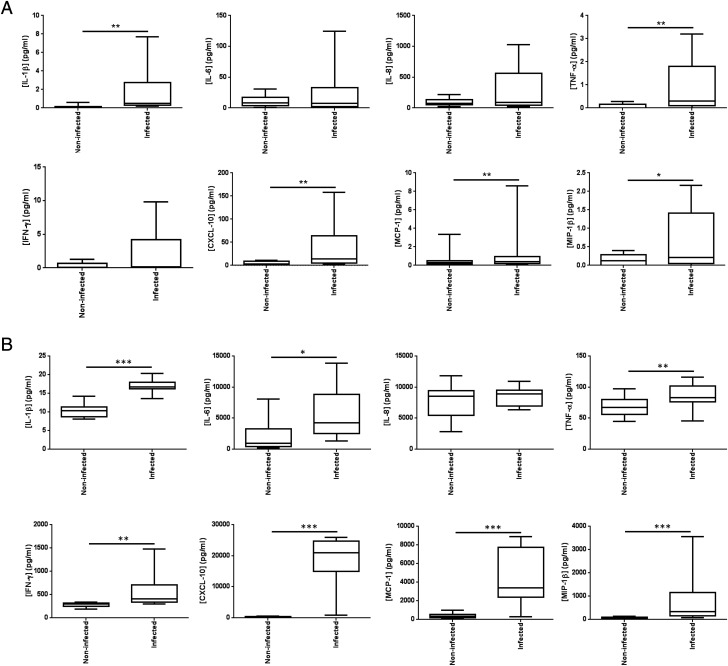
Effect of influenza infection on soluble mediator secretion into the culture medium by (**A**) monolayer primary epithelial cells and (**B**) bronchial tissue explants. PBECs and bronchial tissue explants (Bx) were either mock infected with virus diluent, or infected with influenza virus for 2 h; the virus was then removed and the cells/tissues were incubated for an additional 24 h. The culture supernatants were analyzed by multiplexed ELISA for eight soluble mediators. Data shown are mediator concentrations in pg/ml conditioned medium supernatant. Box and whisker plots indicate median infection-induced mediator concentration and interquartiles ± range, *n* = 9 for PBECs and *n* = 10 for bronchial samples. *p < 0.05, **p < 0.01, ***p < 0.001.

Monolayer PBECs responded to influenza virus infection 24 h postinfection with increased release of Il-1β, CXCL-10, MCP-1, MIP-1β, and TNF-α (Fig. 4). Bronchial explants additionally released IFN-γ and IL-6. The bronchial explants generally secreted 10-fold greater quantities of mediators upon infection, despite containing ∼100-fold fewer epithelial cells, suggesting greater responsiveness to infection of explanted tissues as compared with cell monolayers.

#### Development of a small molecule inhibitor of virus infection.

Vacuolar ATPase was chosen as a target based on its identification as a mediator of early viral replication events in previous studies (8–11). A small molecule vATPase inhibitor, TVB024, was custom synthesized, with a structure based on the indol series of bafilomycin-derived vATPase inhibitors (Supplemental Fig. 2A), but with improved toxicity characteristics (28). Using acridine orange assay, TVB024 was demonstrated to be an effective vATPase inhibitor, as increasing concentrations of the drug inhibited endosome acidification while also reducing influenza infection in a dose-dependent manner (Supplemental Fig. 2B). Its antiviral and cytotoxic profile was validated in monolayer cultures of A549 cells (Supplemental Fig. 2C), in which a 2-log window of therapeutic effectiveness/toxicity could be demonstrated. Data collected in a similar manner using epithelial monolayer and air–liquid interface cultures showed IC_50_ values for TVB024 of 200 and 350 nM, respectively (Table I).

**Table I.** Inhibitor profile for TVB024 using PBECs, air–liquid interface-cultured PBECs (ALI PBEC) and lung tissue explants

Assay	IC_50_	CC_50_
*PBEC*	0.2	>32
*ALI PBEC*	0.35	>32
*Lung tissue explants*	0.88	>10

Samples were pretreated with varying doses of TVB024 for 2 h, and then infected with influenza virus in the presence of inhibitors. Infection was measured by quantitation of viral progeny after a further incubation period of up to 22 h in the presence of inhibitors. Dose-dependent inhibition (IC_50_) calculations were made using GraphPad Prism software by plotting 3-point logistical curves of the percentage of viral release compared with control (uninhibited) in the tissue culture supernatant against the log of the inhibitor concentration. IC_50_ and CC_50_ are half-maximal inhibitor and cytotoxicity concentrations, respectively (values shown are μM).

TVB024 was then compared with oseltamivir carboxylate, the active metabolite of oseltamivir phosphate (a prodrug form of the influenza A and B neuraminidase inhibitor), an established frontline influenza drug recommended for both postexposure treatment and prophylaxis in at-risk patients (7). Optimal doses of 5 μM TVB024 and 100 nM oseltamivir were determined in dose-response experiments of the effects of these inhibitors on viral shedding in lung tissues (Supplemental Fig. 3Bi, 3Bii). For these experiments, surgical resected lungs were used rather than bronchial biopsies because they provide a large enough number of lung parenchymal samples composed of alveolar tissue and small airways. The effect of TVB024 dose on intracellular viral load was confirmed by flow cytometry (Supplemental Fig. 3C), but this confirmation was not sought for oseltamivir, as neuraminidase inhibitors do not affect intracellular viral replication. The optimal doses of TVB024 and oseltamivir showed low levels of toxicity, as measured by LDH release from tissues treated with the compounds (Supplemental Fig. 4).

### Demonstration of antiviral effects of the vATPase inhibitor

#### Effect on infection and inflammatory responses of treatments applied at optimal doses.

**FIGURE 5.**
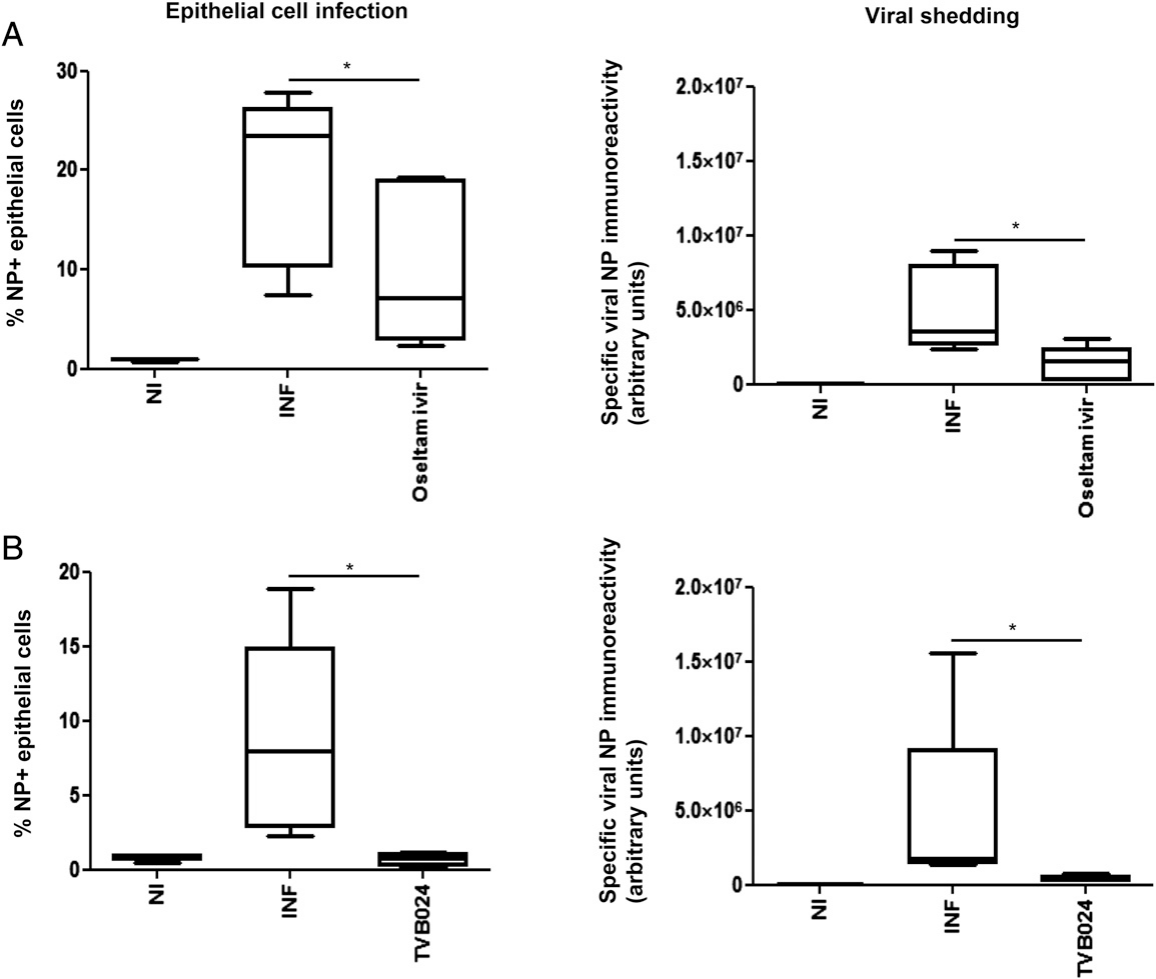
Effect of vATPase and neuraminidase inhibition on viral infection and shedding in bronchial explants. Tissue explants were treated with (**A**) oseltamivir (100 nM) or (**B**) TVB024 (5 μM) for 2 h prior to infection with influenza virus and then incubated for a total of 48 h. Cells dispersed from the tissues were analyzed by flow cytometry for epithelial influenza infection, and culture media were assessed for viral protein immunoreactivity, as previously described. Data shown are mean ± SEM of five experiments. *p < 0.05. INF, infected; NI, not infected.

Having established that 5 μM TVB024 and 100 nM oseltamivir had an optimal inhibitory effect on virus shedding and were not toxic, these doses were applied to bronchial biopsy explants from healthy volunteers. When applied prophylactically (2 h prior to infection), oseltamivir effectively reduced viral shedding but reduced infection only by median 73% (Fig. 5A), presumably by limiting reinfection, as oseltamivir does not inhibit primary infection at this dose. TVB024 was more effective and inhibited epithelial infection by 100% (Fig. 5B), and this was associated with absence of virus shedding.

**FIGURE 6.**
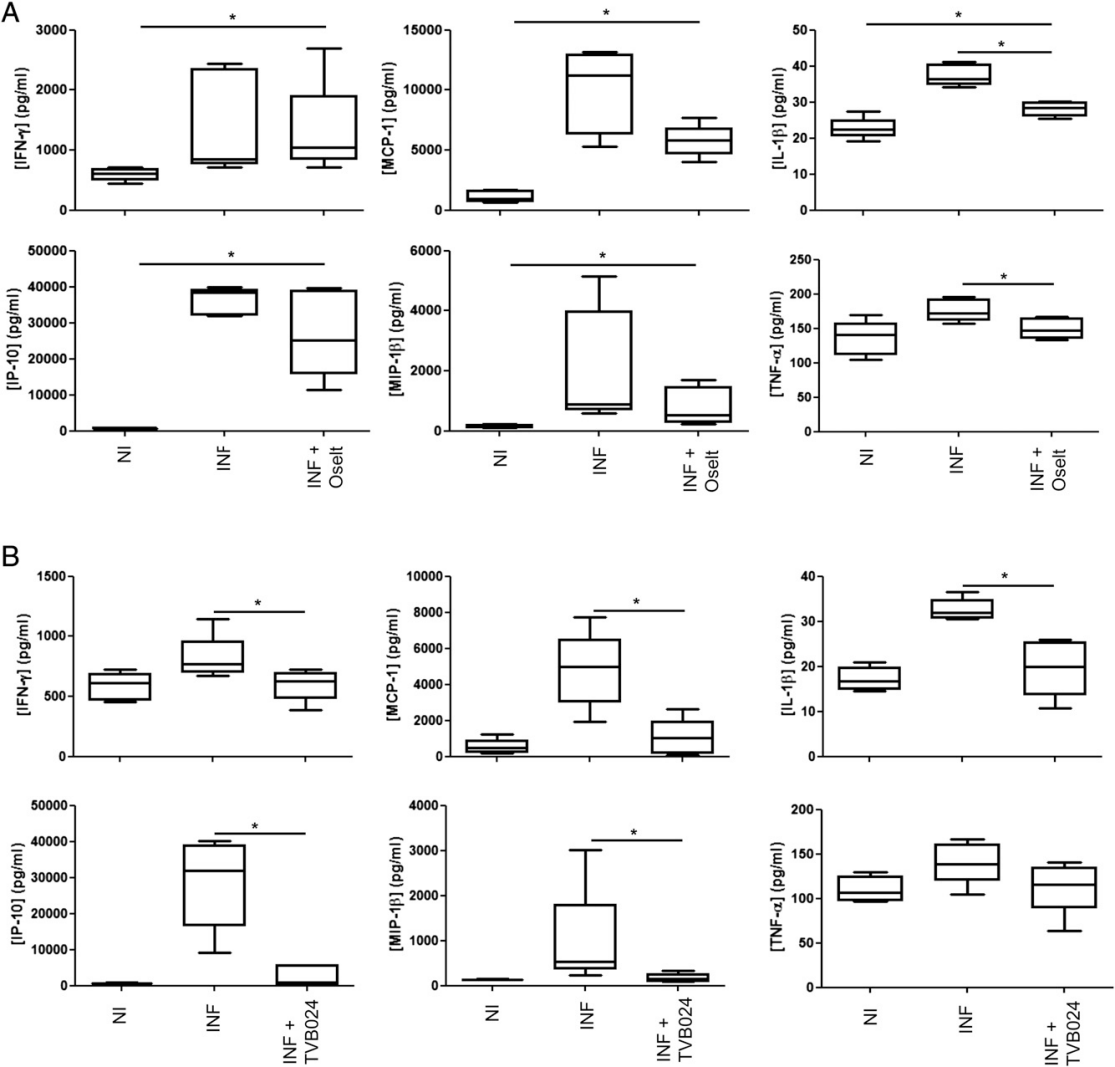
Effect of vATPase and neuraminidase inhibition on cytokine/chemokine release. Tissue-conditioned media were analyzed by multiplexed ELISA to quantify cytokine/chemokine release following prophylactic treatment with (**A**) neuraminidase inhibitor oseltamivir and (**B**) vATPase inhibitor TVB024. Treatment controls (Flu) were prophylactically treated with 0.1% (v/v) DMSO carrier prior to infection. Drug-treated cells were prophylactically treated for 2 h with either 100 nM oseltamivir carboxylate (Flu + Oselt) or 5 μM TVB024 (Flu + TVB024). Box and whisker plots indicate medians and interquartiles ± range, *n* = 5. *p < 0.05. INF, infected; NI, not infected.

To assess how infection-induced inflammatory cytokines and chemokines were affected by these antiviral compounds, mediator release 48 h following infection and antiviral compound treatment of bronchial explants were measured. Despite reducing both viral infection and release, oseltamivir had no effect on the secretion of these mediators, with the exception of IL-1β and TNF-α, which were reduced by ∼50% (Fig. 6A), and only TNF-α was reduced to its baseline level. In comparison, TVB024 treatment (Fig. 6B) significantly reduced five of the mediators, all to baseline levels (IFN-γ, IP-10, IL-1β, MCP-1, and MIP-1β). However, this compound had no significant effect on TNF-α. Neither oseltamivir nor TVB024 significantly reduced infection-induced IL-6 production.

## Discussion

Novel antiviral therapeutics, especially those targeted against host proteins identified by new methods such as small interfering RNA screens, need validation, ideally in a host model. Based on the current study, we propose that bronchial explants offers the opportunity to test new antiviral compounds safely, compare their efficacy to existing drugs, and provide meaningful information on tissue inflammatory responses. To date, epithelial cultures have been used for initial preclinical evidence of efficacy; however, although easily infected with lower doses than needed for tissue explants, PBEC cultures lack the immune cells and structural complexity of intact tissues, resulting in limited inflammatory responses, as shown in this study. Tissue explants have the advantage of a complex architecture without the complicating factors of newly recruited cells or significant adaptive immune components, allowing characterization of primary infection within a complex, yet controlled, system.

Influenza infection of porcine and human lung explants has been demonstrated previously (29, 30), but has relied on immunohistochemical detection of viral nucleoprotein, which poses significant challenges for full quantification. This problem is addressed easily with flow cytometry as applied in our model, enabling robust identification of infection patterns that can be confirmed by immunohistochemistry. A key element of any model is to determine how closely it reflects in vivo processes. We found that influenza virus principally infects epithelial cells in bronchial tissue in line with patterns of infection seen previously in vivo (31).

Influenza-induced inflammation is a significant contributor to morbidity. Studies by others have shown elevation of limited number of inflammatory mediators in blood during influenza infection, including IL-1, IL-6, and IL-8 (32). In the current study, we demonstrate infection-associated upregulation of seven chemokines/cytokines in bronchial tissues. Using explant infections, a more complete array of mediators can be observed than in single-cell cultures, possibly because of the presence of multiple cell types, including macrophages, which are a known source of many of the mediators detected (33).

Previous publications have identified vATPase as a key host gene involved in influenza A infection (8–11), but its role in lung tissue has not been studied to date. To our knowledge, inhibitors of vATPase have not been tested in vivo in humans as antiviral drugs, despite being proposed as a potential target (34). This may be because current vATPase inhibitors, such as clarithromycin and bafilomycin, despite proven in vitro anti-influenza effects (35, 36), are relatively expensive and have unfavorable toxicity profiles. Thus, to our knowledge, our study provides the first conclusive evidence that vATPase inhibition may be an effective way of inhibiting lung infection with influenza virus and the associated inflammatory responses.

Our study suggests that inhibiting host genes involved early in the viral life cycle considerably reduces viral-induced cytokine release, often to background levels. We demonstrated that TVB024 was an effective vATPase inhibitor at concentrations that were not cytotoxic for the duration of the treatment. The concentration range of efficacy of TVB024 was similar to that observed with bafilomycin and concanamycin A (37) but with the added assurance of using a fully synthetic molecule. TVB024 reduced virus release from infected explants more effectively than oseltamivir. Oseltamivir has been extensively studied and shown to be clinically beneficial if taken within 2 d of exposure (38, 39); however, recent meta-analyses have cast its clinical efficacy into doubt. Nevertheless, this study contributes to the understanding of the in vivo mechanisms of oseltamivir. In addition, the study sheds light on the effects of vATPase inhibition. Following viral uncoating within the endosome of the cell and subsequent initiation of replication, virus is detected by pattern recognition receptors such as NOD-like receptors and TLRs. This results in activation of antiviral cascades, which in turn activate proinflammatory cytokine and chemokine release.

The relative efficacies of the two antiviral compounds observed in reducing inflammatory mediator production probably reflect the known mechanisms of action of oseltamivir and vATPase inhibitors. Neuraminidase inhibition reduces viral particle release, but does not affect intracellular viral replication, implying that initial infection rates should not be affected. Thus, neuraminidase inhibition should, theoretically, not have any beneficial effect on proinflammatory mediators. This was, indeed, observed in our study, in which oseltamivir was generally poor at inhibiting infection-induced cytokine release. In contrast, treatment with TVB024 completely inhibited viral shedding and infection-induced cytokine release. This effect would also be observed with other inhibitors targeting viral replication rather than release, irrespective of whether they target viral or host infection mechanisms. However, as previously found with Amantidine (a virally expressed M2 channel inhibitor), viral evasion of this mechanism has been observed, resulting in widespread global resistance (5, 6). Indeed, the influenza strain used in our study is resistant to Amantidine, preventing its use as an early-stage inhibitor control.

Although vATPase inhibition is a more effective anti-influenza agent in human tissues than neuraminidases, in terms of reducing viral replication and its associated inflammation, the physiological effect of inhibition of such a ubiquitously expressed protein remains to be determined. Lung explants can effectively model the direct effects of therapeutic agents on target tissues. However, further toxicological investigations would be needed to examine the systemic effects of such inhibitors in vivo. The proof of efficacy in lung tissues should, however, be the primary initial determinant of anti-influenza therapeutic investigations prior to in vivo toxicology, which is expensive and generally assessed by administering the drug systemically. Localized lung delivery mechanisms could overcome potential systemic toxicity problems that might be overlooked using standard models.

In addition to acting as a bridge between basic research and high throughput screening, on the one hand, and clinical trials with candidate drugs, on the other, our explant model allows safe investigation of infection mechanisms using more hazardous viruses and testing in samples from individuals with pre-existing chronic lung diseases. The donors of bronchial biopsies in this study were healthy, nonatopic nonsmokers <35 y of age, similar to the volunteer profile in clinical trials, but sampling of older patients selected by disease (e.g., asthma or chronic obstructive pulmonary disease, in which morbidity and mortality are greatest) is routinely conducted for research purposes, so it would be easy to extend our study to such at-risk patient populations.

We propose that the lung infection model developed for this proof-of-concept study complements the use of animal models in the discovery of anti-influenza drugs, as it overcomes some of the differences in host–defense mechanisms between humans and other animal species and thereby increases confidence in the efficacy of novel compounds being tested.

In summary, we have provided proof of concept for the use of our ex vivo lung tissue infection model as a clinically relevant, fully human, testing platform for antiviral therapies and have demonstrated the benefits of inhibiting viral replication over viral release in limiting the impact of influenza infections.

## Supplementary Material

Data Supplement
