# Supplementary material for: A Novel Lung Explant Model for the Ex Vivo Study of Efficacy and Mechanisms of Anti-Influenza Drugs
Source: J Immunol. 2015 May 1;194(12):6144–54. doi: 10.4049/jimmunol.1402283 (PMC4456633; doi:10.4049/jimmunol.1402283)
Supplement: Data Supplement [file JI_1402283.zip › JI_1402283_Supplemental_Figures_1.pdf]

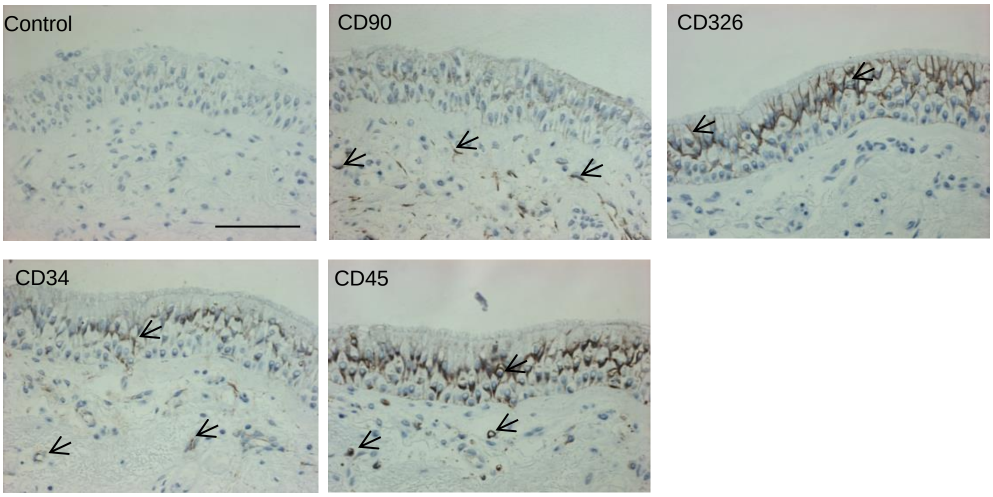

Figure S1 -Immunohistochemical confirmation of monoclonal antibody targets directed against structural and leukocyte cell targets in bronchial explants. GMA embedded bronchial biopsies were cut into 2μm sections and stained using the same monoclonal antibody clones as used for subsequent flow cytometry, directed against fibroblasts (CD90/Thy1, BD clone 5E10), Epithelial cells (EpCAM/CD326, BD clone EBA-1), endothelial cells (CD34, BD clone 8G12) and leukocytes (CD45, Invitrogen clone H130). Detection antibodies were indirectly detected using peroxidase-labelled secondary antibodies and labelled antibodies visualised using DAB stain. Specific immunoreactivity is visible as a brown colouration, haematoxylin counterstain is blue. Size bar indicates 100μm.

A

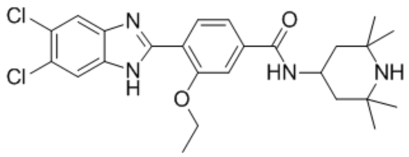

B

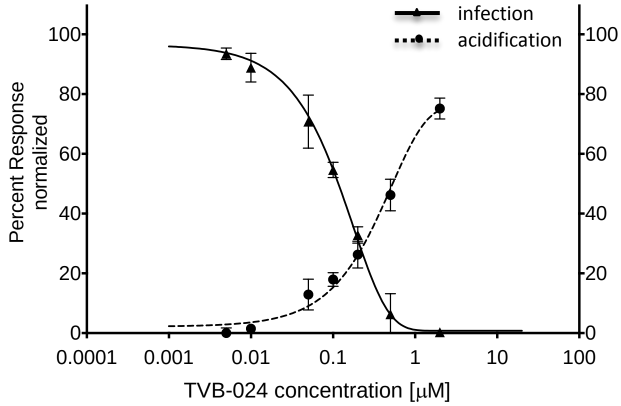

C

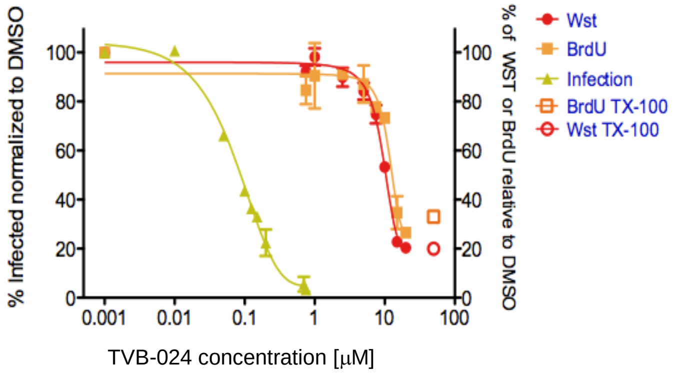

Figure S2 –vATPase inhibitor TVB024 structure and effects on endosomal acidification and cell viability. A) The structure of the TVB024 molecule is based on the indol series of bafilomycin-derived vATPase inhibitors. B) Monolayer cultures of A549 cells were pre-incubated with the TVB024 inhibitor for 2 h prior to infection with X31 influenza for 15h. Cell infection was measured by direct immunofluorescence with a monoclonal antibody directed against the viral NP protein. Infection rates were quantified using image analysis software and expressed on the left y-axis as the % of total cells infected with the virus (green triangles). Viral infection decreased with increasing TVB024 concentration, with full inhibition achieved at 0.5μM. Endosomal acidification measured by acridine orange assay (described in Method section) and expressed as % inhibition, was progressively inhibited by increasing concentrations of TVB024. C) Cellular toxicity induced by the compound was measured by quantifying the loss of cell-associated viability markers BrdU and also water soluble tetrazolium salts (WST) (orange squares and red circles respectively) and expressed as a percentage of DMSO carrier-treated controls. Triton X-100 detergent lysis was used as a 100% toxicity control for the loss of viability and are included on the graph as single data points. Cell toxicity was detected only at concentrations exceeding 5μM, and increased in a concentration-dependent manner. The window of non-toxic maximal efficacy of TVB024 in A549 cells was, therefore, at least one full log scale. Data are mean values from two experiments performed in triplicate, +/- S.D.

A)

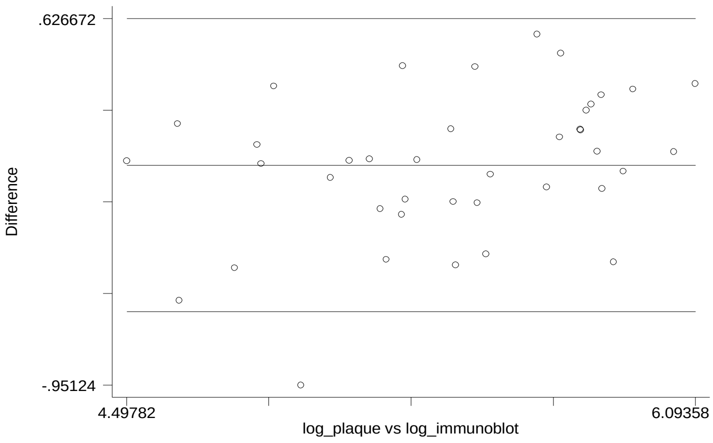

B)

i)

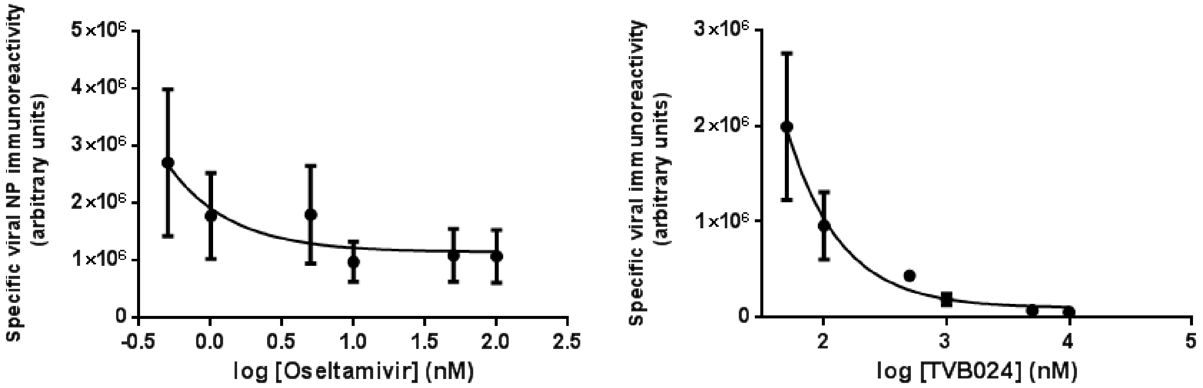

C)

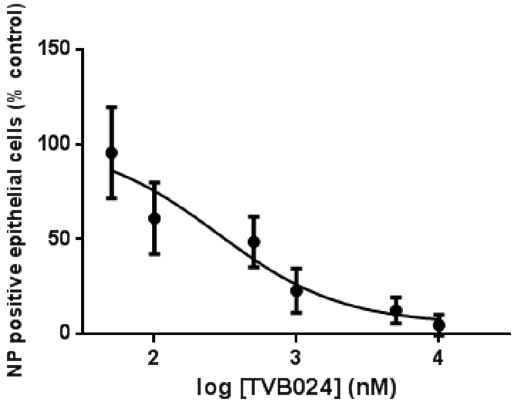

Figure S3 – Validation of viral shedding immunoassay, and dose response curves of anti-viral agents. A) Demonstration of parity in quantitation of viral shedding from influenza-infected lung tissues using MDCK plaque assay, and dot blot immunoassay using polyclonal anti-influenza antibody. Conditioned media samples from bronchial tissues collected 24 h post-infection were analysed by the two methods as described in Materials and Methods. Data were analysed using Bland-Altman comparison of the difference between the two measurements after $\log_{10}$ transformation of the data, showing good agreement between the two methods. B) Dose-response of Oseltamivir and TVB024 inhibition of influenza infection. Lung parenchymal tissue from surgical resections was cultured *ex vivo* as described in the Material and Methods, and pre-treated for 2 h with varying doses of oseltamivir (100-0nM) or TVB024 (10-0μM). Tissue was then infected with log 7.4 infectious unit dose (IUD) A/H3N2/Wisconsin/68/2005 virus for 2 h. The tissue was incubated for a further 48 h in the presence of the inhibitors. Conditioned medium was analysed for viral release into the culture media by immunoblot and results expressed as arbitrary units; this demonstrated a dose-dependent reduction in viral release from infected tissue for both inhibitors. However, whereas TVB024 reduced release to baseline (ii), oseltamivir only reduced it by approximately 50% (i). The IC_{50} for TVB024 and Oseltamivir were 88.3nM and 5nM respectively. The tissue was enzymatically dispersed in order to stain epithelial cells for influenza infection and analysis by flow cytometry (C); this showed that TVB024 reduced infection to baseline in a dose-dependent manner, mirroring the viral release patterns. Data are mean values from three experiments performed in triplicate, +/- S.E.M.

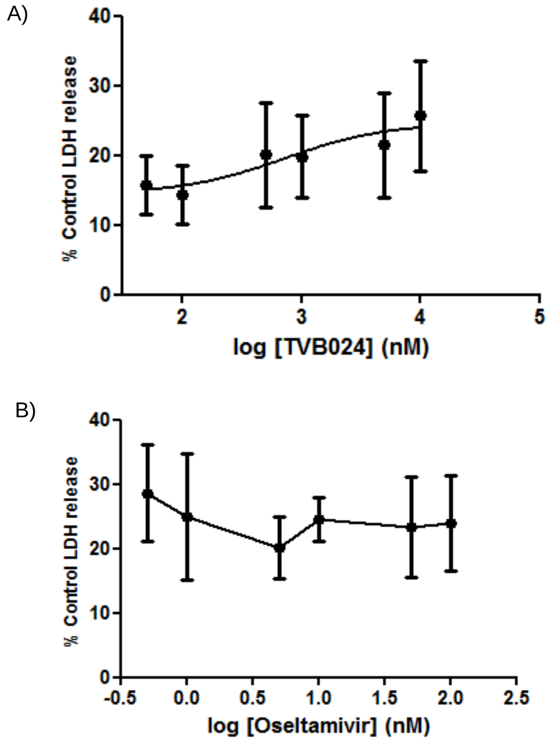

Figure S4 –Dose-response of Oseltamivir and TVB024, effect on LDH release into culture medium as a measure of cytotoxicity. Lung parenchymal tissue from surgical resections was cultured *ex vivo* as described in the Material and Methods, and pre-treated for 2 h with varying doses of oseltamivir (100-0nM) or TVB024 (10-0μM). Tissues were mock infected with virus diluent and then incubated in culture medium for a further 48 h. Culture medium samples were centrifuged at 400g to remove cellular material, and then assayed for LDH concentration using a non-radioactive LDH assay kit (Promega) according to the manufacturer's instructions. Positive control samples (100% LDH) were generated by homogenising tissue samples in culture medium. Data are expressed as mean percentage of positive control LDH values +/- S.E.M from three experiments performed in triplicate. No significant alteration in LDH release above baseline values was detected following drug treatments using these inhibitor concentrations.
